# Identifying therapeutic biomarkers of zoledronic acid by metabolomics

**DOI:** 10.3389/fphar.2023.1084453

**Published:** 2023-04-25

**Authors:** Xiang Li, Zi-Yuan Wang, Na Ren, Zhan-Ying Wei, Wei-Wei Hu, Jie-Mei Gu, Zhen-Lin Zhang, Xiang-Tian Yu, Chun Wang

**Affiliations:** ^1^ Department of Osteoporosis and Bone Diseases, Shanghai Clinical Research Center of Bone Disease, Shanghai Sixth People’s Hospital Affiliated to Shanghai Jiao Tong University School of Medicine, Shanghai, China; ^2^ Clinical Research Center, Shanghai Sixth People’s Hospital Affiliated to Shanghai Jiao Tong University School of Medicine, Shanghai, China

**Keywords:** zoledronic acid, osteoporosis, metabolomics, biomarkers, RNA-Seq, ovariectomy, rats

## Abstract

Zoledronic acid (ZOL) is a potent antiresorptive agent that increases bone mineral density (BMD) and reduces fracture risk in postmenopausal osteoporosis (PMOP). The anti-osteoporotic effect of ZOL is determined by annual BMD measurement. In most cases, bone turnover markers function as early indicators of therapeutic response, but they fail to reflect long-term effects. We used untargeted metabolomics to characterize time-dependent metabolic shifts in response to ZOL and to screen potential therapeutic markers. In addition, bone marrow RNA-seq was performed to support plasma metabolic profiling. Sixty rats were assigned to sham-operated group (SHAM, n = 21) and ovariectomy group (OVX, n = 39) and received sham operation or bilateral ovariectomy, respectively. After modeling and verification, rats in the OVX group were further divided into normal saline group (NS, n = 15) and ZOL group (ZA, n = 18). Three doses of 100 μg/kg ZOL were administrated to the ZA group every 2 weeks to simulate 3-year ZOL therapy in PMOP. An equal volume of saline was administered to the SHAM and NS groups. Plasma samples were collected at five time points for metabolic profiling. At the end of the study, selected rats were euthanatized for bone marrow RNA-seq. A total number of 163 compound were identified as differential metabolites between the ZA and NS groups, including mevalonate, a critical molecule in target pathway of ZOL. In addition, prolyl hydroxyproline (PHP), leucyl hydroxyproline (LHP), 4-vinylphenol sulfate (4-VPS) were identified as differential metabolites throughout the study. Moreover, 4-VPS negatively correlated with increased vertebral BMD after ZOL administration as time-series analysis revealed. Bone marrow RNA-seq showed that the PI3K-AKT signaling pathway was significantly associated with ZOL-mediated changes in expression (adjusted-p = 0.018). In conclusion, mevalonate, PHP, LHP, and 4-VPS are candidate therapeutic markers of ZOL. The pharmacological effect of ZOL likely occurs through inhibition of the PI3K-AKT signaling pathway.

## 1 Introduction

Postmenopausal osteoporosis (PMOP) is a prevalent disease and is a major cause of death and disability in elderly individuals (Cummings and Melton, 2002; Compston et al., 2019; Johnston and Dagar, 2020; Liu et al., 2022). The prevalence of osteoporosis among women aged 40 years and older is as high as 20.6% in China, and the incidence of osteoporosis and fracture risk increase with age (Wang L. et al., 2021). Zoledronic acid (ZOL) is a front-line anti-resorptive agent that increases bone mineral density (BMD) and reduces fracture risk (Black et al., 2007; Camacho et al., 2020). The therapeutic effects of ZOL are typically verified by annual BMD measurement. In addition, bone turnover markers (BTMs) such C-terminal telopeptide of type I collagen and procollagen type I N-peptide are early indicators of therapeutic effects because significant changes occur within the first few months of treatment. However, the serum levels of BTMs do not fluctuate with prolonged treatment time, nor can they predict the long-term treatment effects or fracture risk (Black et al., 2012; Black et al., 2015). Furthermore, no indicators have been identified to aid in treatment plan selection, predict adverse drug reactions, predict the risk of new fractures, aid in determination of when patients enter the “bisphosphonate drug holiday”, and when to restart treatment (Naylor and Eastell, 2012; Eastell and Szulc, 2017).

We used untargeted metabolomics in an *in vivo* model to understand the metabolic changes that occur in response to ZOL treatment, and to screen potential biomarkers related to efficacy. In addition, bone marrow RNA-seq was performed to explore changes in tissue-specific expression following ZOL treatment.

## 2 Materials and methods

### 2.1 Reagents

Zoledronic Acid Injection (100 ml : 5 mg) was purchased from Jiangsu Chia Tai-Tianqing Pharmaceutical CO. (Jiangsu, China). Estradiol II electrochemiluminescence kit was purchased from Roche (Risch-Rotkreuz, Switzerland). Methanol, acetonitrile, formic acid, ammonium formate, ammonium hydroxide, and ammonium bicarbonate were purchased from Sigma-Aldrich (Missouri, United States). RNA integrity was assessed using the RNA Nano 6000 Assay Kit for the Bioanalyzer 2100 system (Agilent Technologies, CA, United States). TRIZOL (Thermo Fisher, Massachusetts, United States).

### 2.2 Animal model and experimental design

This study was conducted in accordance with the guidelines of the Laboratory Animal Center of Shanghai Jiao Tong University. Sixty female four-week-old Sprague Dawley rats were purchased from Charles River and housed in a specific pathogen-free, well-ventilated environment, with 12-h light/dark illumination cycles at a constant temperature of 24°C ± 2°C and humidity of 40%–60%. Animals were allowed free access to food and water. The experiment extended from 4 weeks to 12 months of age. Nine months of age was marked as week 0, and every single week afterward was marked as week 1, week 2 till week 12 (12 months of age), sequentially.

**FIGURE 1:**
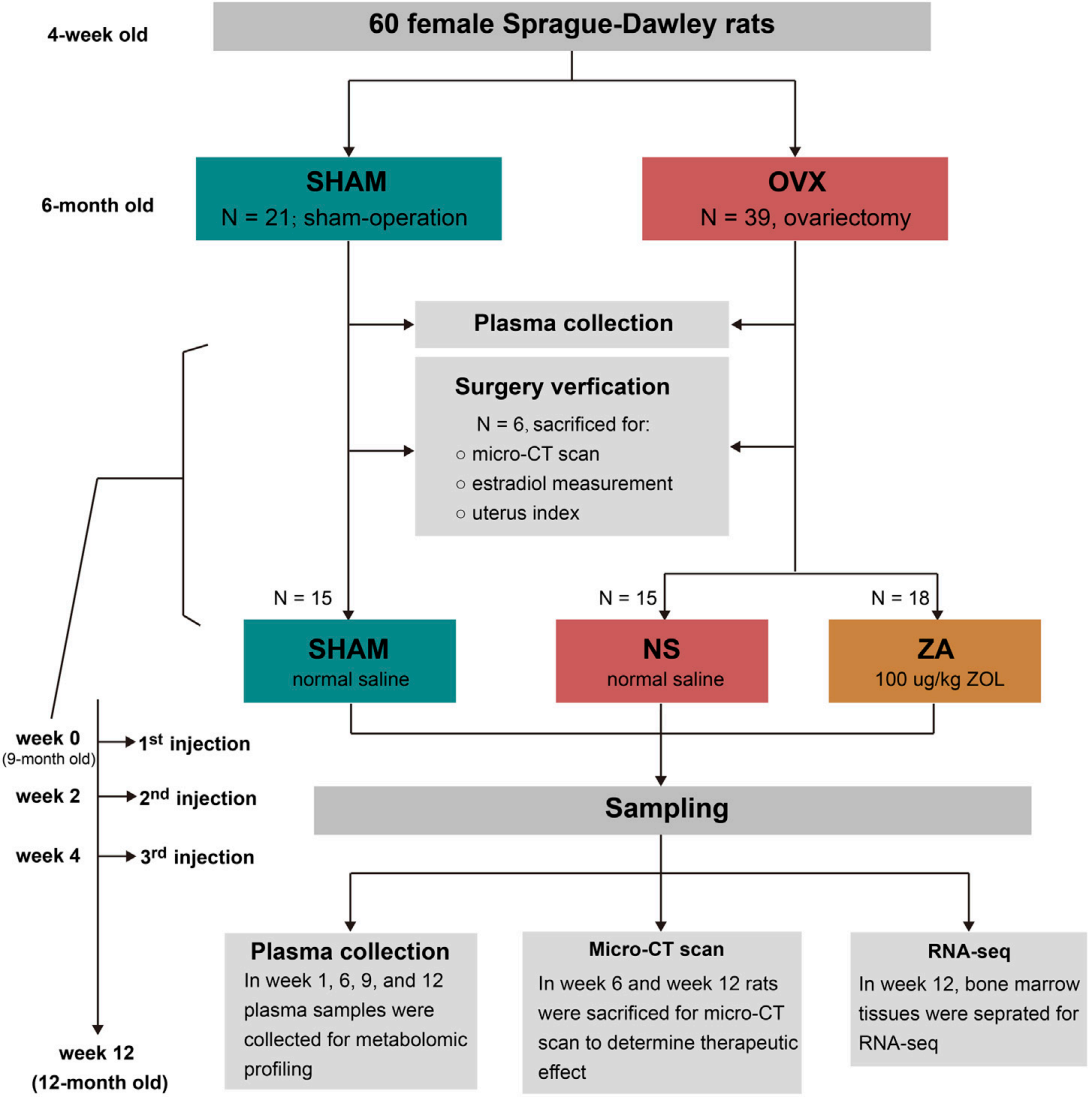
Illustration of the study design.

The study design is shown in Figure 1. All rats were randomly divided into sham-operated (SHAM, n = 21) and ovariectomy (OVX, n = 39) groups at 6-month old, and received sham operation or bilateral ovariectomy, respectively. After operation, the animals were housed until 9 months of age, and six rats in each group were selected and euthanized to verify the modeling. Then, the remaining 33 rats in the OVX group were divided into ZOL group (ZA, n = 18) and normal saline group (NS, n = 15). Rats in the ZA group were given 100 μg/kg ZOL subcutaneously at week 0, week 2, and week 4 to simulate 3-year ZOL therapy in patients with PMOP. The same volume of normal saline was given to the SHAM and NS groups. At weeks 0, 1, 6, 9, and 12, plasma samples were collected for metabolomic profiling. At weeks 6 and 12, rats from each group were selected and euthanatized for micro-computed tomography (μ-CT) analysis to assess the therapeutic effects of ZOL. In addition, bone marrow tissues were separated for RNA-seq at week 12.

### 2.3 Estradiol measurement and uterus index calculation

At week 0, six rats selected from the OVX and SHAM groups were euthanatized by carbon dioxide asphyxiation followed by immediate cardiac puncture. Two ml of blood was collected and silenced for 30 min. The blood was then centrifuged at 1400 × g for 10 min at 4°C, and the supernatant was collected and frozen at −80°C. The uterus was excised and weighed.

To verify model success, serum samples were evaluated for estradiol using electrochemiluminescence. The uterus index was calculated by dividing the wet weight of the uterus (mg) by the body weight (g).

### 2.4 Bone micro-structure assessment by μ-CT

At weeks 0, 6 and 12, the right femurs and the second lumbar (L2) vertebrae of euthanatized rats were collected for determination of volumetric bone mineral density (vBMD) using micro-CT (Bruker Corporation, Kontich, Belgium). NRecon software Version 1.6 (Bruker Corporation, Kontich, Belgium) was used for three-dimensional reconstruction and image visualization.

### 2.5 Untargeted metabolomics

The rats were fasted but had free access to water overnight before sampling at weeks 0, 1, 6, 9, and 12 for metabolomics profiling. After isoflurane inhalation anesthesia, 400 μl of blood was extracted from the periorbital venous plexus and placed a centrifuge tube containing EDTA for 10 min. The tube was then centrifuged at 1400 × g for 10 min at room temperature. The plasma was stored at −80°C.

#### 2.5.1 Sample preparation

Plasma was thawed in ice bath and vortexed for 10 s. Two-hundred μl of sample was extracted in 800 μl of methanol. The mixtures were shaken for 3 min and precipitated by centrifugation at 4000 × g for 10 min at 20°C. Four aliquots of 100 μl supernatant were transferred to sample plates and dried under blowing nitrogen, then reconstituted for ultra-performance liquid chromatography/tandem mass spectrometry (UPLC-MS/MS) analysis. The UPLC-MS/MS systems were previously described (Shen et al., 2020).

#### 2.5.2 Compound identification and quantification

After quality control and pre-processing, ion peaks were extracted. Metabolites were identified by searching an in-house library generated from running reference standards purchased or obtained from other sources. Metabolite identification required strict matching of three criteria between experimental data and library entries: narrow window retention index, accurate mass with variation less than 10 ppm, and MS/MS spectra with high forward and reverse searching scores. The peak area for each metabolite was calculated using area-under-the-curve. The normalized peak areas were then log-transformed. Missing values in the peak area matrix were imputed by using the minimal detection value of a metabolite among all samples.

#### 2.5.3 Data processing

Analysis was performed using R (R 3.4.1) and Rstudio (1.4.1717). The R packages mixOmics, pROC, and Mfuzz were used. Multivariate analyses such as principal component analysis (PCA) and orthogonal partial least square discriminant analysis (OPLS-DA) were performed. Time-series analysis was performed to determine dynamic metabolic changes. Statistical discrepant metabolites were determined using the Wilcox rank test. Variable importance of projection (VIP) > 1 and *p* < 0.05 were used for screening differential metabolites. Differential metabolites were further analyzed using metabolite set enrichment analysis (MSEA) on MetaboAnalyst 5.0 (https://www.metaboanalyst.ca/).

### 2.6 RNA-seq and multi-omics integrated analysis

At week 12, three rats from each group were chosen for transcriptomic analysis. After euthanasia, bone marrow tissues were separated as previously described (Bai et al., 2020). Bone marrow cells (5 × 10^5^) were collected and pipetted with 1 ml of TRIZOL. The mixture was frozen at −80°C.

After sample and library preparation and sequencing, differential expression analysis was performed using the DESeq2 R package (1.20.0). The read counts were adjusted using the edgeR program package through one scaling normalized factor prior to differential gene expression analysis. Differential expression analysis was performed using the edgeR R package (3.22.5). *p* < 0.05 and absolute fold change of 2 were set as the thresholds for screening differentially expressed genes. We used clusterProfiler R package to conduct pathway enrichment analysis in Kyoto Encyclopedia of Genes and Genomes (KEGG). The transcriptional and metabolic data were integrally analyzed.

### 2.7 Statistics

For non-omics statistics, independent-samples t-test was used for comparisons between two groups. Analysis of variance was used for comparisons among three groups. Statistical significance was defined as *p* < 0.05. Data are expressed as the mean ± standard deviation. We used SPSS 22.0 (IBM, NY, United States) for statistical analysis and GraphPad Prism 8 software (GraphPad Software Inc., CA, United States) for data visualization.

## 3 Results

### 3.1 Modeling verification

At week 0, the success of modeling was verified by serum estradiol measurement and uterus index calculation. The serum estradiol level in the SHAM group was 50.4 ± 12.3 pmol/L. In contrast, estradiol levels were lower than the test range in the OVX group. The uterus index in the SHAM group was significantly higher than that in the OVX group (1.899 ± 0.500 *vs*. 0.451 ± 0.116 mg/g, *p* < 0.001).

### 3.2 Body weight and vBMD at baseline and follow-up

**FIGURE 2:**
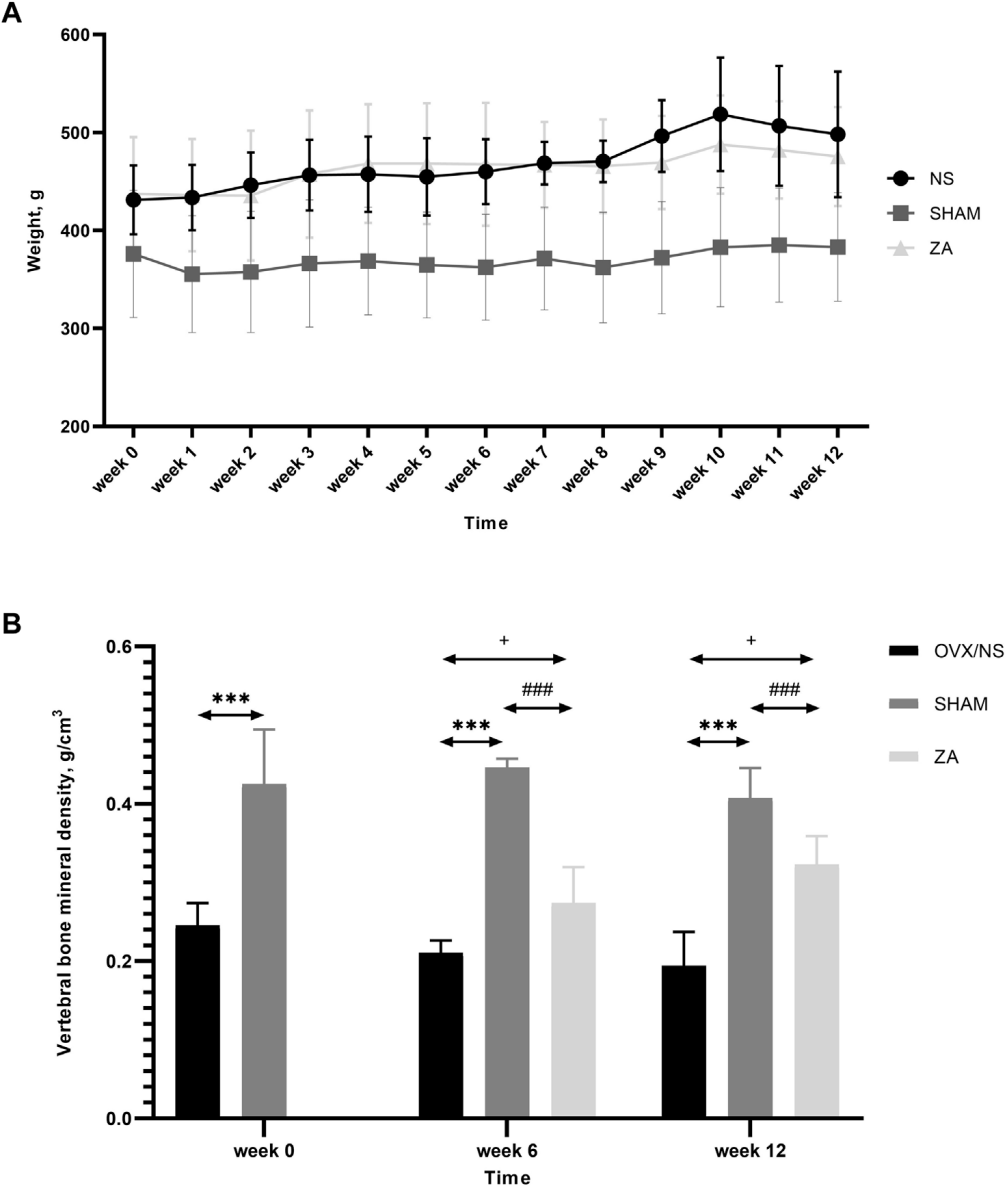
Body weights and volumetric bone mineral density (vBMD) of the second lumbar (L2) vertebrae. **(A)** Body weights of rats at different time points. Each value is expressed as the mean ± SD. Significant differences were observed in the SHAM *versus* NS and ZA groups at every time point, but no differences were observed between NS and ZA groups (determined using two-way ANOVA). **(B)** vBMD of L2 vertebrae. ^***^ *p* < 0.001 SHAM *versus* NS; ^###^ *p* < 0.001 SHAM *versus* ZA;+<0.05 ZA *versus* NS.

Body weight was significantly lower in the SHAM group than that in the NS and ZA groups throughout the course of the study. Body weight did not differ between the NS and ZA groups (Figure 2A). In addition, the vBMD of L2 vertebrae in the SHAM group was the highest among the three groups throughout the course of the study (Figure 2B).

**FIGURE 3:**
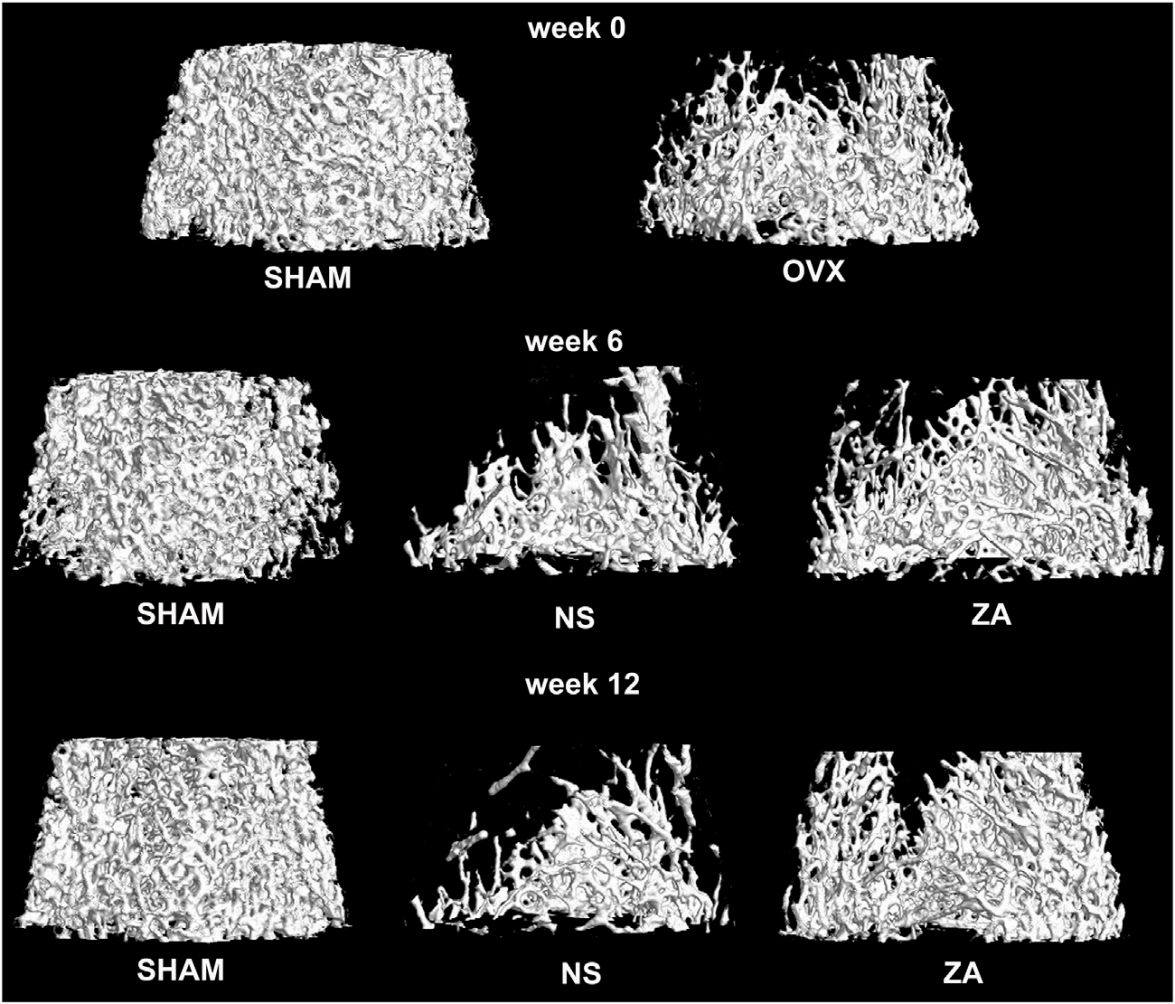
Three-dimensional reconstruction of the second lumbar vertebrae.

At baseline, significantly higher vBMD was found in SHAM group than in OVX group (Figure 2B). At week 6, the vBMD of L2 vertebrae in the ZA group was significantly higher than those in NS group (0.273 ± 0.039 g/cm^3^ *vs*. 0.210 ± 0.011 g/cm^3^, *p* = 0.031). At week 12, this significant difference continued to increase (0.322 ± 0.030 g/cm^3^ *vs*. 0.194 ± 0.036 g/cm^3^, *p* = 0.002). Three-dimensional reconstruction of vertebrae is shown in Figure 3.

The femoral trabecular vBMD of SHAM group were 0.073 ± 0.020 g/cm^3^, 0.088 ± 0.001 g/cm^3^, 0.080 ± 0.011 g/cm^3^ at baseline, week 6, and week 12, significantly higher than the ZA and NS groups throughout the study (Supplementary Figure S1A). Femoral cortex vBMD was significantly higher in the SHAM group than that in the NS group at week 12 (0.392 ± 0.003 g/cm^3^ *vs*. 0.380 ± 0.002 g/cm^3^, *p* = 0.001), but not at any other time point (Supplementary Figure S1B).

### 3.3 Metabolic profiling

**FIGURE 4:**
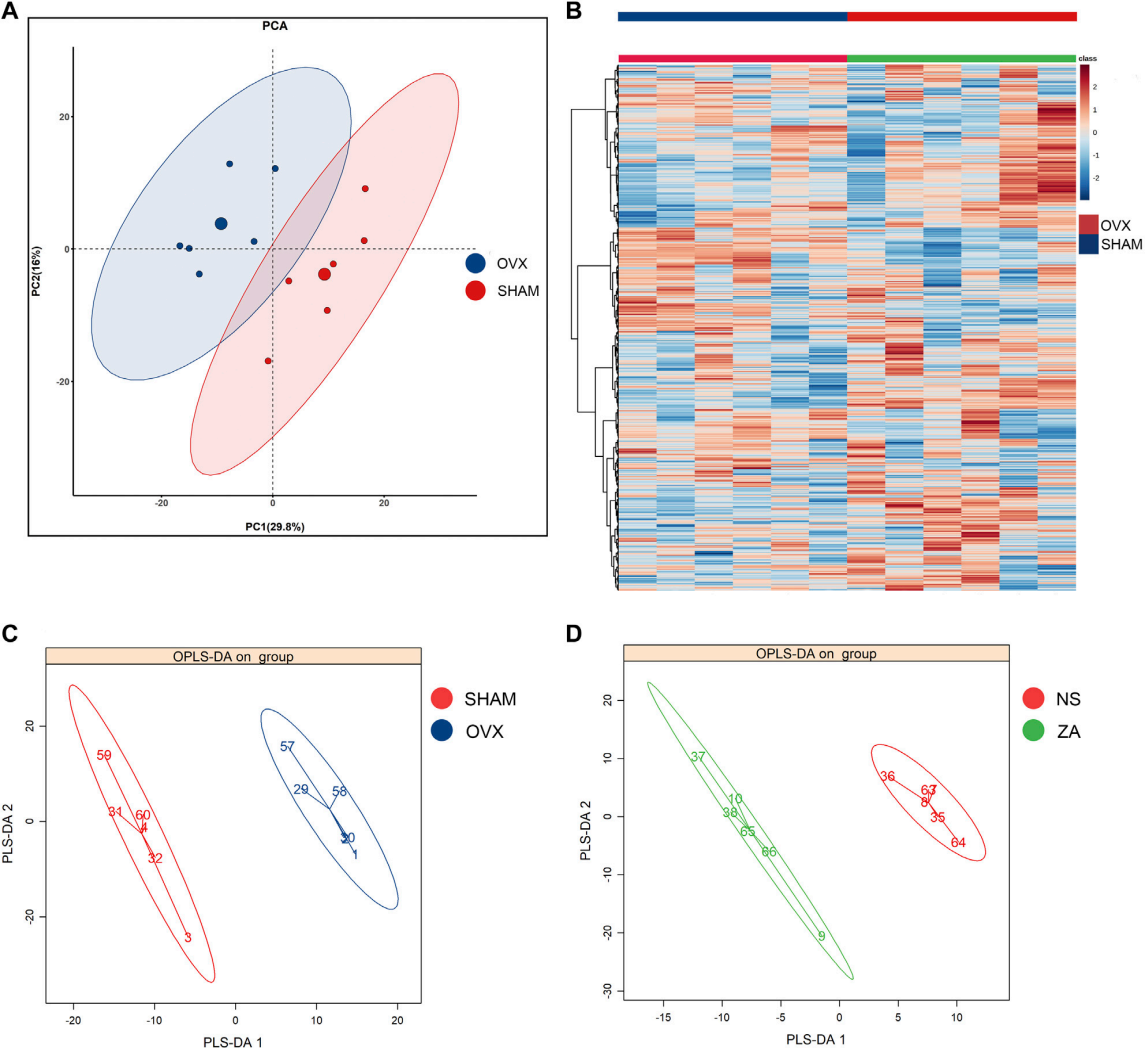
Multivariate statistical analysis of metabolic profile. **(A)** PCA score plot of OVX *versus* SHAM at week 0; **(B)** heatmap of metabolites in OVX *versus* SHAM at week 0; **(C, D)** OPLS-DA score plot of OVX *versus* SHAM at week 0 and ZA *versus* NS at week 1.

Untargeted metabolomics was performed using previously described UPLC-MS/MS platform. A pooled QC sample was generated by pooling all samples. The QC sample was injected multiple times during the testing sequence. The signal of internal standards was stable, with a median relative standard deviation of 3.14%. After raw data pre-processing and ion peak extraction, metabolites were identified based on RI, accurate mass, and MS/MS spectra. The peak area of each metabolite was calculated and normalized. A total of 786 metabolites were detected and relatively quantified in our study. The data were then analyzed using PCA, which is an unsupervised approach. The metabolomic profiles of the NS and SHAM groups were significantly different at week 0 (Figure 4A). A heatmap based on different metabolites is shown in Figure 4B. The remaining PCA plots are shown in Supplementary Figure S2. There were no significant separations between the ZA and NS groups in PCA (Supplementary Figure S3). We then performed OPLS-DA supervised methodology, to identify important differential metabolites. The score plot of OPLS-DA showed clear separations at the different time points between the OVX/NS and SHAM groups (Figure 4C; Supplementary Figure S4A–D), and between the ZA and NS groups (Figure 4D; Supplementary Figure S4E–G). VIP was used to further screen differential metabolites. The reliability of OPLS-DA models was reflected by the parameters R2 (cum) and Q2 (cum). The reliability of each model is summarized in Supplementary Table S1.

### 3.4 Differential metabolite screening and metabolic pathway analysis

**FIGURE 5:**
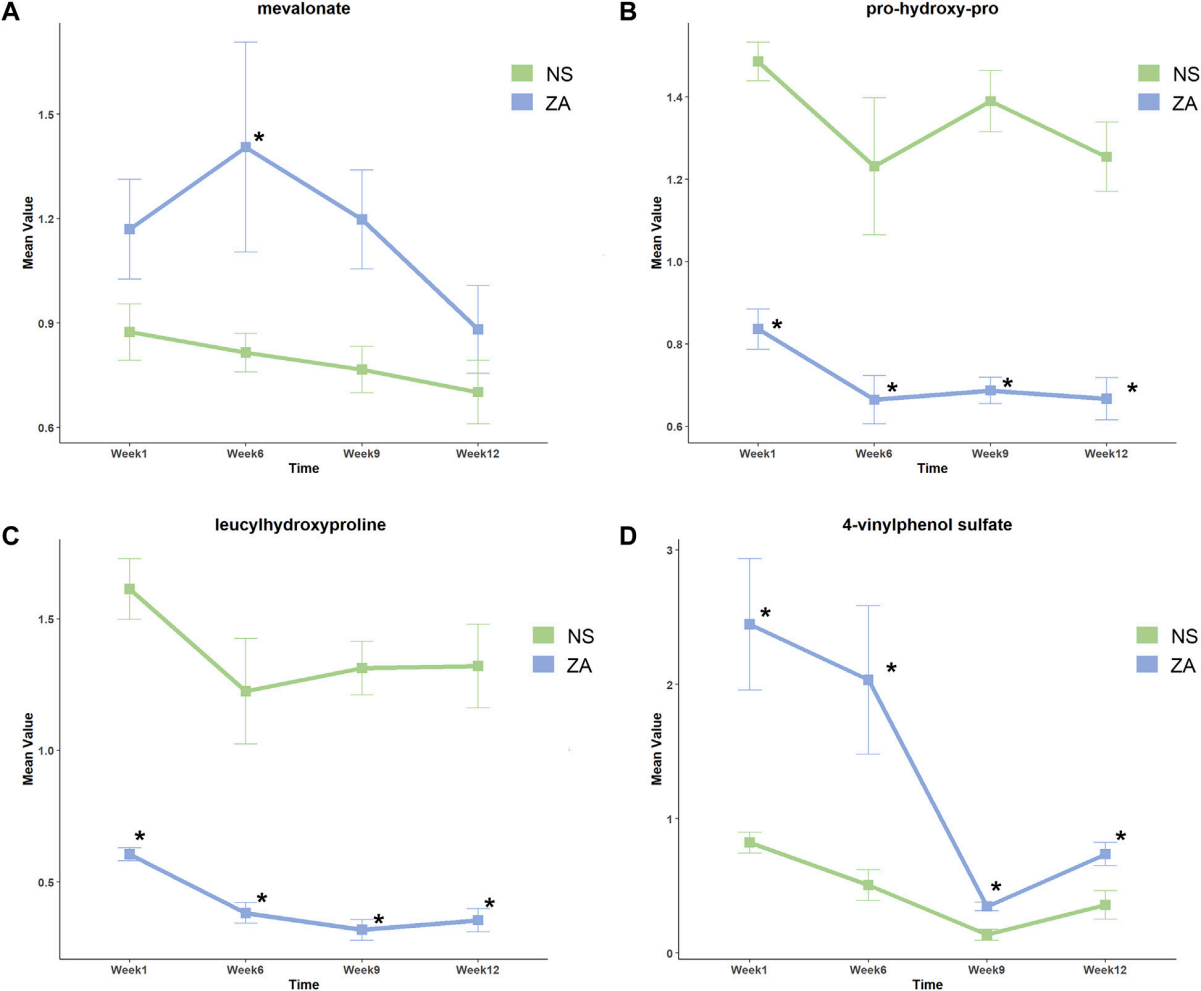
Relative changes in key metabolites in the ZA and NS groups at different time points. **(A)** Mevalonate, **(B)** prolyl hydroxyproline, **(C)** leucyl hydroxyproline, and **(D)** 4-vinylphenol sulfate. *Identified as differential metabolites.

VIP >1 and *p* < 0.05 as determined by the Wilcox rank test were used to screen differential metabolites. The time-dependent metabolic fingerprints were determined by differential metabolites at different time points (Supplementary Material). Twenty-six metabolites including pseudouridine, and metabolites associated with glutamate and glutathione metabolism, tryptophan metabolism, and arginine metabolism differentially abundant between the NS and SHAM groups at every time point. Basic information of the 26 pivotal metabolites is shown in Supplementary Table S2. A total of 163 differential metabolites were identified between the ZA and NS groups (Supplementary Material). Among these differential metabolites, pro-hydroxy-pro (prolyl hydroxyproline, PHP), leucyl hydroxyproline (LHP), and 4-vinylphenol sulfate (4-VPS) were differentially abundant at all four time points. In addition, mevalonate, a key molecule of the pathway through which ZOL exerts pharmacological action, was identified as a differential metabolite at week 6. Basic parameters regarding the four pivotal metabolites and their relative changes are summarized in Table 1; Figure 5.

**TABLE 1:** Intersections of differential metabolites (ZA *versus* NS).

Compound name	Week 1			Week 6			Week 9			Week 12		
	Mean ratio	p	VIP	Mean ratio	p	VIP	Mean ratio	p	VIP	Mean ratio	p	VIP
prolyl hydroxyproline	0.563	0.005	3.226	0.540	0.045	1.884	0.494	0.005	2.926	0.531	0.005	2.992
leucyl hydroxyproline	0.375	0.005	3.332	0.312	0.045	2.086	0.242	0.005	2.903	0.268	0.005	3.1534
4-vinylphenol sulfate	2.977	0.005	2.740	4.026	0.030	1.714	2.549	0.013	2.382	2.055	0.031	2.041

VIP, variable importance of projection.

**FIGURE 6:**
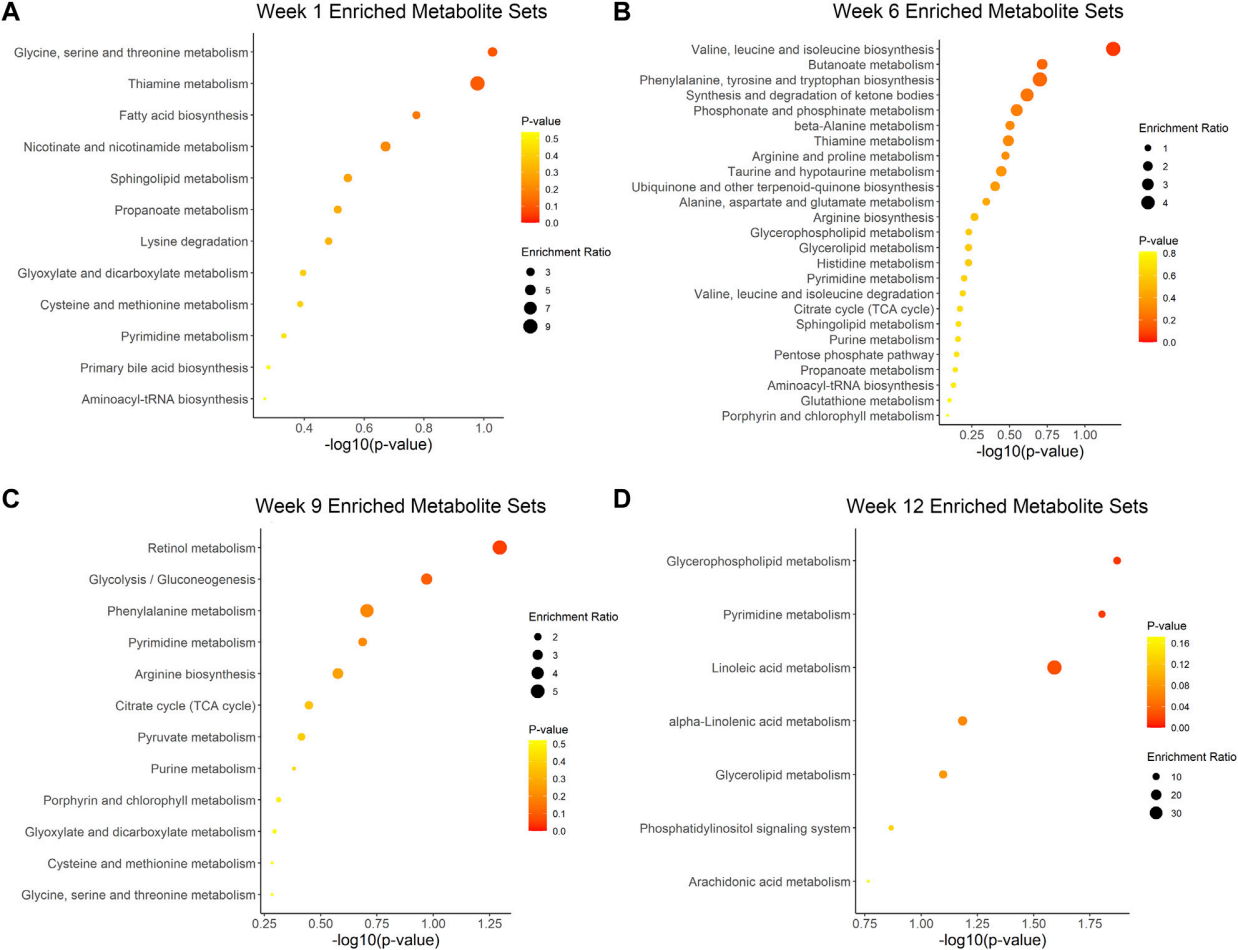
Metabolic pathway enrichment analysis of ZA *versus* NS. Enriched pathways at week 1 **(A)**, week 6 **(B)**, week 9 **(C)**, and week 12 **(D)**.

We further described the major pathways affected by ovariectomy and ZOL administration using MSEA. Aminoacyl-tRNA biosynthesis, glycine-serine-threonine metabolism, phenylalanine-tyrosine-tryptophan biosynthesis, and arginine biosynthesis metabolites sets were significantly enriched at different time points, but they were not significant at each time point (Supplementary Figure S5). No pathways differed significantly between the ZA and NS groups at week 0, week 6 or week 9 (Supplementary Material, Figures 6A–C). At week 12, glycerophospholipid metabolism, pyrimidine metabolism, and linoleic acid metabolism pathways were significantly different between the ZA and NS groups (Figure 6D), with *p* values of 0.01, 0.02, and 0.03, respectively.

### 3.5 Time-series analysis

**FIGURE 7:**
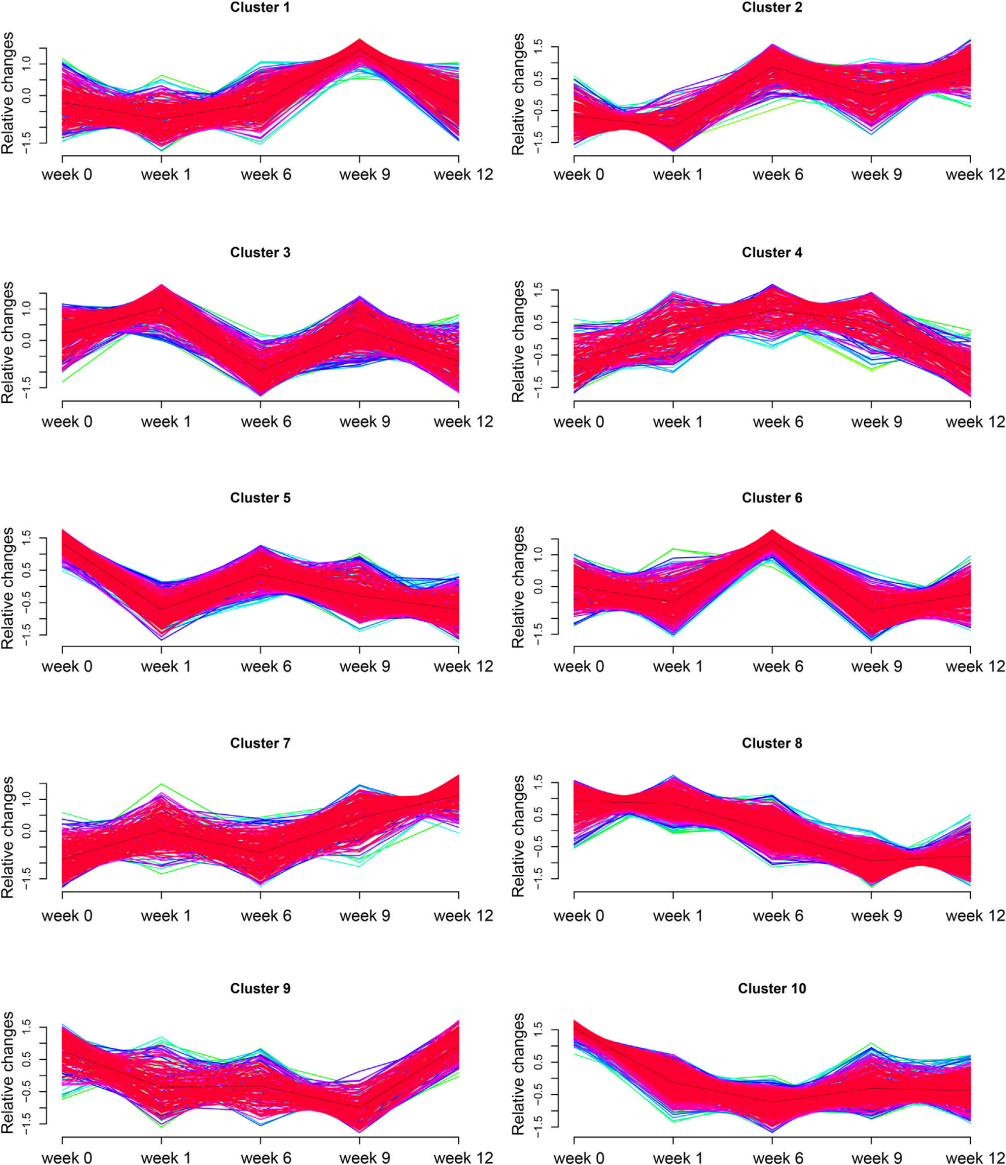
Time-series analysis. Ten clusters of metabolites with time-dependent metabolic pattern alterations.

To further screen valuable metabolites, Mfuzz was used for time series analysis to determine dynamic changes and clustering of metabolites. Ten clusters of metabolites with unique changes were categorized (Figure 7). Among all clusters, cluster 8 showed a unique trend in which it was negatively correlated with increasing vertebral vBMD after ZOL intervention. We compared all cluster 8 metabolites between differential metabolites regarding ZA group *versus* NS group. An intersection of 34 metabolites was identified (Supplementary Material). Notably, 4-VPS, a component of cluster 8, remained differentially abundant throughout the experiment.

### 3.6 RNA-seq and multi-omics integrated analysis

**FIGURE 8:**
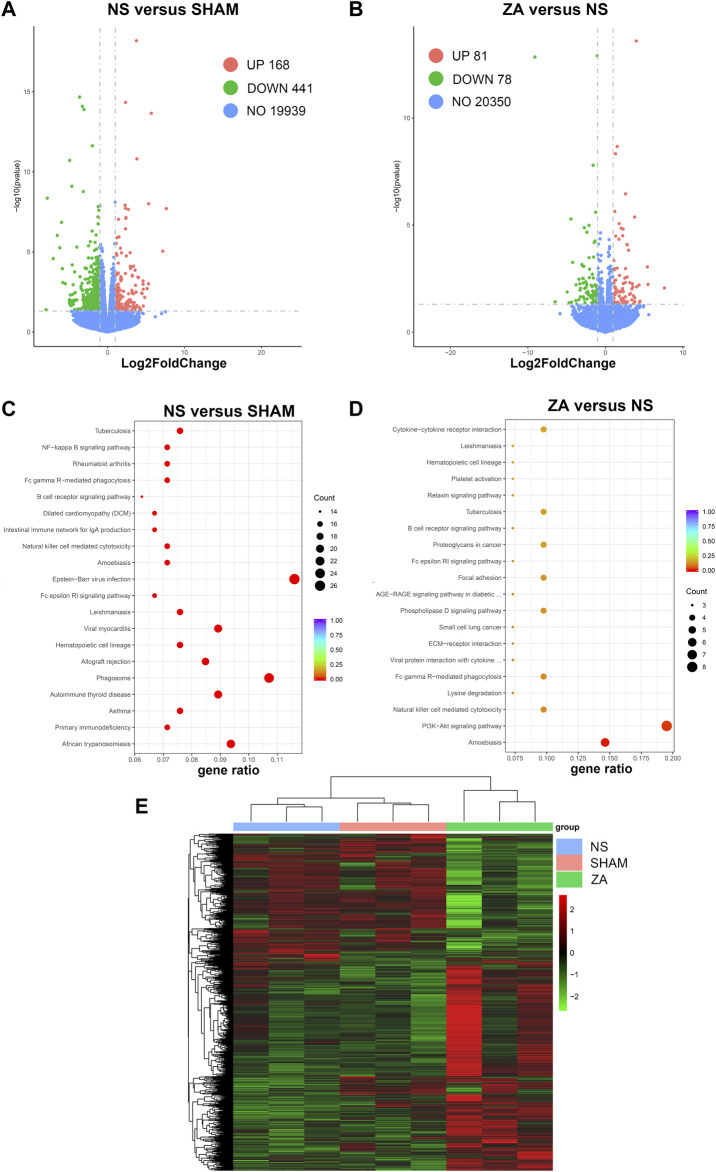
Transcriptomic differential expression analysis. Differentially expressed genes are illustrated using volcano plots: **(A)** NS *versus* SHAM, **(B)** ZA *versus* NS. KEGG pathway enrichment analysis: **(C)** NS *versus* SHAM; **(D)** ZA *versus* NS. **(E)** Heatmap with color changes to highlight changes in expression among the NS, SHAM, and ZA groups.

Bone marrow cell RNA-seq was performed following standard protocols. Data were validated after stringent quality control. After reference genome mapping and quantification of expression, differential analysis was performed. Differentially expressed genes between the NS and SHAM groups and between the ZA and NS groups are shown as volcano plots (Figures 8A, B). A heatmap was generated to highlight expression changes among all three groups (Figure 8E). Then, we performed KEGG pathway enrichment analysis of differentially expressed genes (DEGs). (Figures 8C, D).

Enrichment analysis resulted in identification of 33 KEGG pathways with adjusted *p* < 0.05 between NS and SHAM. Taking gene ratio (GR) and biological significance into account, natural killer cell mediated cytotoxicity (GR = 16/224), Fc gamma R-mediated phagocytosis (GR = 16/224), and NF-kappa B signaling pathway (GR = 16/224) were potentially associated with the pathogenesis of PMOP. In ZA *versus* NS, 41 DEGs were identified and matched to the KEGG database. In the pathway enrichment analysis, the PI3K-AKT signaling pathway was significantly enriched, with an adjusted-p value of 0.018 and background ratios of 437/10534. 19.5% of DEGs were matched to the PI3K-AKT signaling pathway, including the genes that encode type I collagen—*Col1a1* and *Col1a2*. Therefore, the PI3K-AKT signaling pathway was regarded as a pivotal pathway that correlates with the pharmacological actions of ZOL. The remaining RNA-seq findings are included in the Supplementary Material.

Integrated omics resulted in the identification of thermogenesis and phospholipase D signaling pathways as the only differentially enriched pathways between the NS and SHAM groups, though few metabolites were matched (Supplementary Material). No significant pathways were identified between the ZA and NS groups.

## 4 Discussion

Estrogen deficiency is the primary mechanism of PMOP. Estrogen deficiency reduces estrogen-mediated osteoclast apoptosis and leads to increased expression of NF-kappa B ligand (RANKL) in bone marrow stromal cells (Rachner et al., 2011). In this study, a PMOP model was successfully generated by bilateral ovariectomy, as shown by uterus index, serum estradiol levels, and baseline vertebral vBMD. Using this animal model, we confirmed that ZOL treatment increased the trabecular vBMD of lumbar vertebrae in ovariectomized rats. The vertebral vBMD in the ZA group increased 6 weeks after the first administration of ZA and continued to increase throughout the study compared to that in the NS group. Successful modeling allowed for further omics analysis to identify ZOL-induced changes in PMOP and screen therapeutic biomarkers.

Metabolomics is a high-throughput technology that comprises advanced analytical chemistry techniques and sophisticated statistical methodologies. This tool characterizes metabolism in organism through identification of various metabolites (Nicholson and Lindon, 2008). This allows for identification of biomarkers with potential prognostic and diagnostic value. Several studies have identified potential diagnostic biomarkers of osteoporosis using metabolomics (You et al., 2014; Moayyeri et al., 2018; Wang et al., 2019; Mei et al., 2020). However, few studies have focused on biomarkers related to treatment response. Plasma metabolomics can be used to characterize overall metabolism of an organism, but cannot be used to characterize cell- or tissue-specific metabolism. Tissue-specific RNA-seq can be used to aid in identification of important biological pathways. Integrated metabolomics and transcriptomics have been used to characterize diseases like Alzheimer’s disease, liver injury, and diabetes (Li et al., 2020; Mahajan et al., 2020; Ma et al., 2021). However, hardly any attempts have been made in osteoporosis research with the help of multi-omics methodology.

In this study, time-dependent metabolic shifts were characterized using untargeted metabolomics. In the NS *versus* SHAM groups, 26 compounds associated with glutamate metabolism, tryptophan metabolism, and arginine metabolism were identified as critical metabolites. Pseudouridine is the most abundant RNA epigenetic modification in living organisms and plays an active role in modification of non-coding RNA and mRNA (Li et al., 2016; Guzzi et al., 2018). Our finding suggested that epitranscriptomics may contribute to the pathogenesis of PMOP. Arginine and its metabolites play a role in bone health. In primary osteoblasts and osteoblast cell lines, L-arginine promotes type I collagen synthesis via activation of the growth hormone-insulin-like growth factor-1 axis (Chevalley et al., 1998). In addition, L-arginine facilitate nitric oxide production to inhibit bone resorption (Visser and Hoekman, 1994; Fini et al., 2001). Treatment with RANKL increased protein arginine methyltransferase 1 (PRMT1) expression and promoted osteoclastogenesis, but its resorption-promoting effect was attenuated after silencing PRMT1 (Choi et al., 2018). Tryptophan influences bone metabolism through its derivates serotonin, melatonin, and kynurenine. Serotonin originating from the gut inhibits bone formation, and brain-derived serotonin has been shown to promote bone formation and suppress resorption (Michalowska et al., 2015). Melatonin acts on melatonin receptor 2 to facilitate osteoblast proliferation, differentiation, and mineralization (Sharan et al., 2017). Kynurenine is associated with osteoblastogenesis and plays a key role in ameliorating bone-aging phenotypes (Al Saedi et al., 2020; Anaya et al., 2020). Glutamate and glutathione (GSH) metabolism also act on bone metabolism. Neural glutamate transporters, such as the glutamate/aspartate transporter, are expressed by osteoblasts and osteocytes. In addition, many glutamate receptors are expressed by bone cells (Morimoto et al., 2006; Lee et al., 2021). Yuan et al. (2022) showed that GSH concentrations in ovariectomized mice were lower than those in the sham-operation group. In a subsequent study, GSH was proved to interfere with osteoclast energy metabolism through inactivation of the cAMP response element-binding protein pathway.

The current study showed time-dependent metabolic changes by revealing differential metabolites and associated pathways in response to ZOL treatment. Analysis using MSEA did not identify any consistently enriched pathways, which indicated that the pharmacological action of ZOL was time dependent. Mevalonate, a critical component of the pathway targeted by ZOL, was differentially abundant at week 6. PHP, LHP, and 4-VPS were differentially abundant at each time point. Therefore, these metabolites were potential biomarkers of therapeutic effects.

The antiresorptive effect of nitrogen-containing bisphosphonates (NBPs) occurs through inhibition of farnesyl pyrophosphate synthetase (FPPS) in the mevalonate pathway (Reszka and Rodan, 2003). Inhibition of FPPS leads to a decrease in farnesyl pyrophosphate. This change results in increased levels of mevalonate through negative feedback (Goldstein and Brown, 1990). Our study showed significantly higher mevalonate levels in the ZA group at week 6, which was consistent with the assumed variation resulting from FPPS inhibition. Inhibition of FPPS by ZOL was dose- and time-dependent, which may explain why mevalonate levels did not differ at all time points. Our previous study of alendronate, an oral NBPs medication, showed that genetic variations in *MVK* (encodes mevalonate kinase) were associated with BMD response after treatment (Wang et al., 2015). These findings suggest that mevalonate is a potential biomarker of therapeutic response. Mevalonate may also be associated with adverse effects of ZOL. Lack of osteoblasts potentially causes osteonecrosis of jaw. Zafar et al. (2016) showed that ZOL induced apoptosis of primary alveolar osteoblasts by interfering with the mevalonate pathway. Elevated serum creatinine was observed in some patients with PMOP who were treated with ZOL (Black et al., 2007). A preclinical study showed that the nephrotoxicity of ZOL might result from FPPS inhibition in kidney and tubular cells (Lühe et al., 2008). According to the changing trend of the mevalonate level in this study, we suggested that the mevalonate level increased immediately after ZOL treatment and decreased with time. However, the relationship between this change, bone mineral density, and bone micro-architecture changes remains to be clarified.

PHP and LHP were differentially abundant throughout the study, which indicated a strong relationship between bone metabolism and hydroxyproline (HP). HP is produced by post-translational modification of proline in collagen, and comprises 35%–40% of bone matrix (Belostotsky and Frishberg, 2022). Urinary HP correlates with collagen synthesis and is regarded as an index of bone turnover. In patients with Paget’s disease of the bone, urinary HP is markedly reduced after calcitonin treatment (Johnston, 1972), which indicates that HP is a potential biomarker for antiresorptive therapy. PHP shows greater clinical potential than HP because it is not affected by diet and is abundant in human peripheral blood (Husek et al., 2008; Inoue et al., 2016). A clinical study showed that administration of alendronate for 3 months resulted in decreased urinary PHP levels. Urinary PHP decreased further after 6 months of treatment, then increased slightly at 9 months (Husek et al., 2008). Several fundamental studies have shown that PHP promotes osteoblasts differentiation and upregulates the osteogenic gene *RUNX2* by disrupting the interaction between RUNX2 and FOXG1 (Kimira et al., 2014; Kimira et al., 2017; Nomura et al., 2019; Nomura et al., 2021). PHP and LHP showed almost consistent variations in our study. Compared with the NS group, PHP and LHP were significantly lower since week 1; a marked decline was seen at week 6, and remained low thereafter. The unique changes in PHP and LHP were in line with the changes in BTMs after ZOL administration (Black et al., 2007), potentially indicating an inhibited bone turnover.

In the present study, 4-VPS was the only metabolite that was identified as a critical metabolite and showed a unique negative correlation with increased vBMD in the ZA group. This finding indicated that 4-VPS may be associated with ZOL treatment response. 4-Vinylphenol sulfate is a critical metabolite of 4-vinylphenol (4-VP), a xenobiotic derived from styrene metabolism. Styrene is widely used in the plastic industry, and is also an environmental contaminant in food, tobacco smoke, and engine exhaust (Manini et al., 2003). 4-VP is conjugated and excreted as either a glucuronide or sulfate (4-VPS) (Manini et al., 2002). An epigenome-wide association study showed that 4-VPS was associated with methylation of *RARA*, which participates in regulation of cell development, differentiation, and apoptosis (Petersen et al., 2014). A Mendelian randomization study showed that one standard deviation increase in 4-VPS levels was associated with a 22% higher risk of developing heart failure (Wang Z. et al., 2021). Therefore, we suppose 4-VPS might also be an indicator of adverse effects. Since 4-VPS is a xenobiotic and animals were nourished in the same environment, it is speculated that ZOL impacted enzymes of styrene metabolism and led to the increase of 4-VPS in plasma.

Our transcriptomic data showed that the PI3K-AKT pathway was disturbed after ZOL administration. The PI3K-AKT signaling pathway plays a critical role in bone metabolism and is associated with molecules that are important in bone homeostasis, such as parathyroid hormone, Wnt3a, and calcium-sensing receptor. Furthermore, the PI3K-AKT pathway is also strongly associated with the classic Wnt and NF-kappa B pathways (Marie, 2012; Amjadi-Moheb and Akhavan-Niaki, 2019). In osteoclastogenesis, AKT activates osteoclast differentiation by triggering the GSK3β/NFAtc1 signaling cascade (Moon et al., 2012). PI3Kγ promotes osteoclastogenesis and induces bone loss in mice (Kang et al., 2010). The PI3K-AKT pathway may also play a key role in the pathogenesis of osteoporosis. Previous studies have shown that miR-140-3p inhibits *PTEN* expression. Yin et al. (2020) discovered that higher miR-140-3p levels and lower PTEN levels were detected in the peripheral blood mononuclear cells (PBMCs) of an osteoporosis cohort than those in the control cohort. Transfection of a miR-140-3p inhibitor inhibited proliferation and differentiation and promoted apoptosis in PBMCs, which suggested that the PTEN/PI3K-AKT signaling pathway may play a key role in osteoporosis. In glucocorticoid-induced osteoporosis, glucocorticoids enhance osteoclast autophagy through PI3K-AKT/mTOR inhibition (Fu et al., 2020; Li et al., 2021; Xing et al., 2021). Alendronate significantly inhibits AKT phosphorylation and phosphatidylinositol 3,4,5-trisphosphate (a product of PI3K activation) production (Inoue et al., 2005). ZOL promotes reactive oxygen species generation and inhibits PI3K and AKT phosphorylation, thus strengthening the anti-tumor effect of cisplatin in osteosarcoma (Liu et al., 2021). In RAW264.7 cells, mildronate and alendronate impede osteoclast formation by decreasing phosphorylation of extracellular signal-regulated kinase 1/2 and AKT (Tsubaki et al., 2014). These findings indicate that the anti-osteoporotic effects of ZOL likely occur through the PI3K-AKT pathway. The expressions of *Col1a1* and *Co1a2*, the genes that encode type I collagen, were found to be downregulated in the ZA group, indicating a slower bone turnover rat after ZOL administration.

We used metabolomics and transcriptomics to characterize biochemical markers and biological pathways associated with ZOL treatment, which highlighted the potential for use of multi-omics methodology to study osteoporosis. Our study was subject to the following limitations. First, metabolic profiling is performed using non-targeted metabolomics, so the absolute concentrations of metabolites was not determined. Second, RNA-seq and metabolomics were not completely synchronized, which limits conclusions based on integrated analysis. Finally, we did not perform further characterization of the four critical metabolites identified in this study.

## 5 Conclusion

This study showed that metabolic changes caused by ovariectomy and ZOL administration were time dependent. Mevalonate, PHP, LHP, and 4-VPS were potential biomarkers of ZOL treatment efficacy. The PI3K-AKT signaling pathway actively participated in ZOL-induced expressional changes, and may be critical to the anti-osteoporosis effects of ZOL. Our findings provided a basis for identification of novel therapeutic markers and emphasized the prospect of multi-omics methodology in osteoporosis research.

## Data Availability

Raw data of bone marrow RNA-seq can be found here: https://www.ncbi.nlm.nih.gov/search/all/?term=PRJNA904509.
