# Supplementary material for: Identifying therapeutic biomarkers of zoledronic acid by metabolomics
Source: Front Pharmacol. 2023 Apr 25;14:1084453. doi: 10.3389/fphar.2023.1084453 (PMC10166846; doi:10.3389/fphar.2023.1084453)
Supplement: Supplementary file 1 [file DataSheet6.docx]

Supplementary Material

**Identifying therapeutic biomarkers of zoledronic acid by metabolomics**

**Xiang Li^1,†^, Zi-yuan Wang^1,†^, Na Ren^1^, Zhan-ying Wei^1^,Wei-wei Hu^1^, Jie-mei Gu^1^, Zhen-lin Zhang^1^, Xiang-tian Yu^2^****^,^* and Chun Wang^1,^***

*** Correspondence:** Xiang-tian Yu^2,*^graceyu1985@163.com; ChunWang^1,^*wangchun66@sjtu.edu.cn

^†^ **Equal contribution and first authorship:** Xiang Li^1,†^, Zi-yuan Wang^1,†^ contributed equally to this work and share first authorship

# Supplementary Data

Relevant data of the current study were uploaded as supplementary files. The description of each file was as follows:

"Differential_metabolites.xlsx" Differential metabolites of NS versus SHAM and ZA versus NS; Differential and differential metabolites of ZA in cluster 8;

"MSEA.xlsx" Metabolite sets enrichment analysis regarding OVX/NS versus SHAM and ZA versus NS.

"Integrative_analysis.xlsx" The results of integrative analysis of metabolomics and transcriptomics;

“RNAseq_NS_vs_SHAM.xlsx” RNA-seq results of the NS group versus the SHAN group.

“RNAseq_ZA_vs_NS.xlsx” RNA-seq results of the ZA group versus the SHAM group.

# Supplementary Figures and Tables

## Supplementary Figures

_
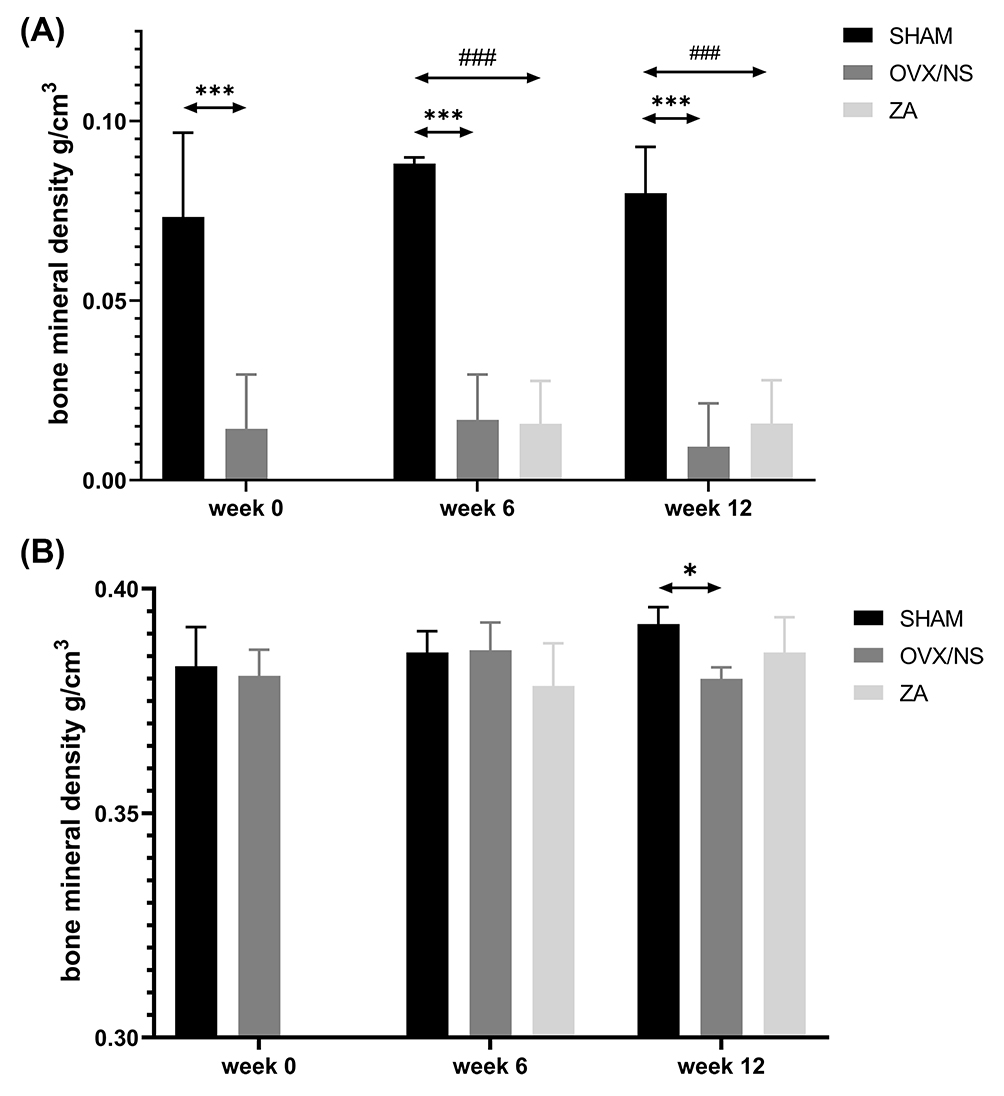
_

**Supplementary Figure S1.** Volumetric bone mineral density (vBMD) of the right femur. (A) Trabecular vBMD. ^***^p ＜0.001, SHAM versus OVX/NS; ^###^p ＜0.001, SHAM versus ZA. (B) Cortical vBMD. ^*^p ＜0.05, SHAM versus NS.


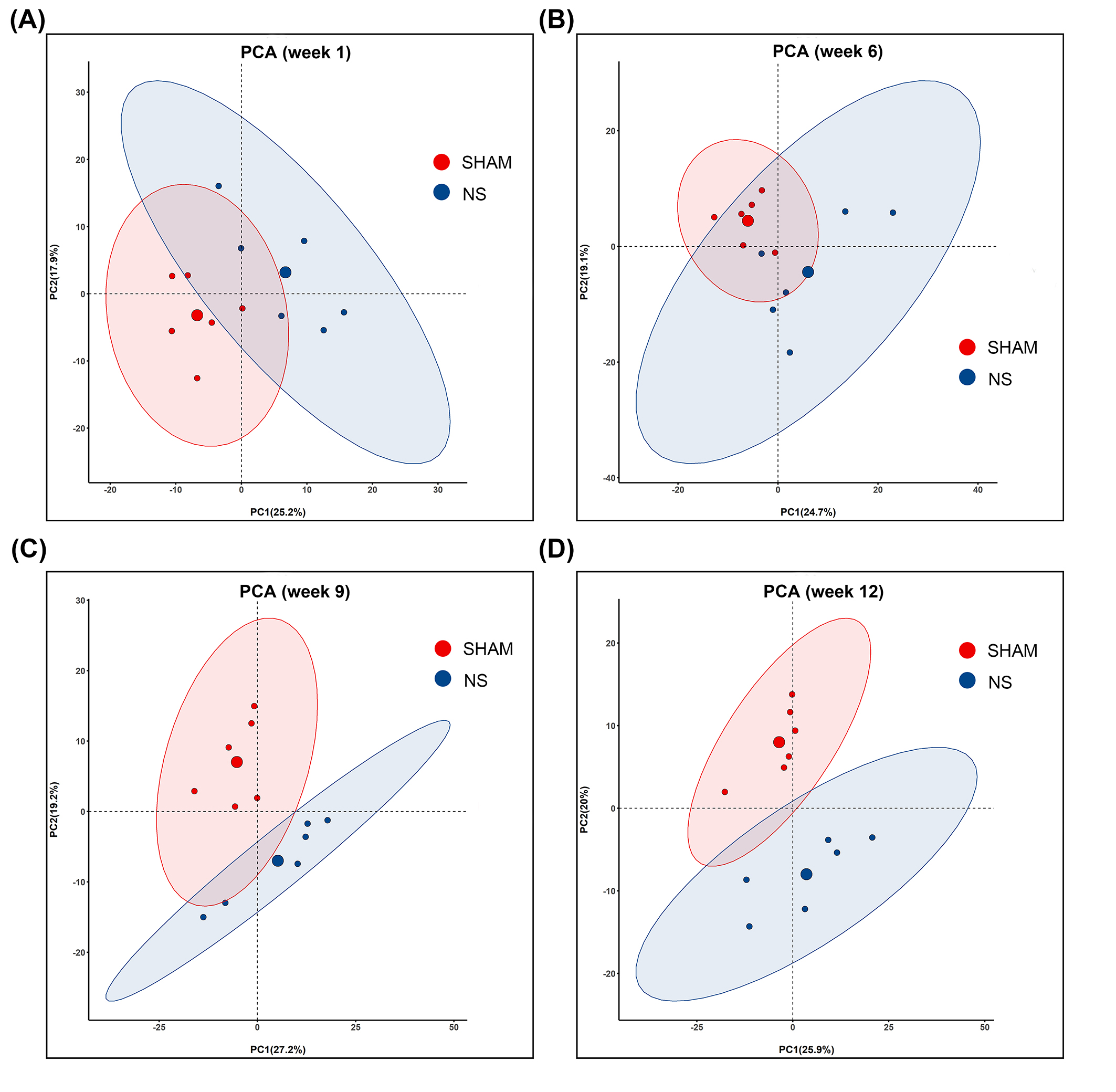


**Supplementary Figure S2.** PCA score plots of NS versus SHAM at week 1 (A), week 6 (B), week 9 (C), and week 12 (D).


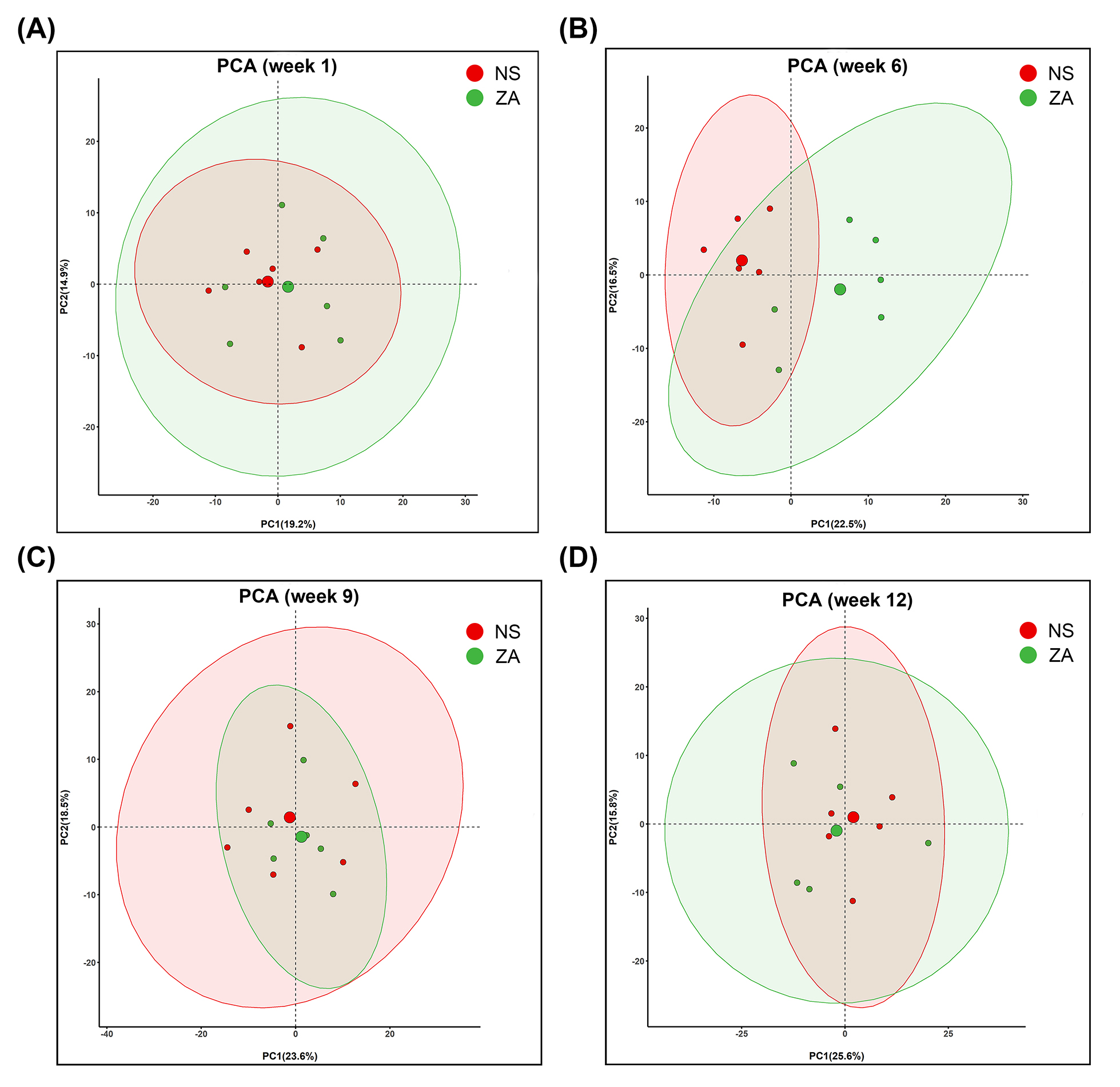


**Supplementary Figure S3.** PCA score plots of ZA versus NS at week 1 (A), week 6 (B), week 9 (C), and week 12 (D).


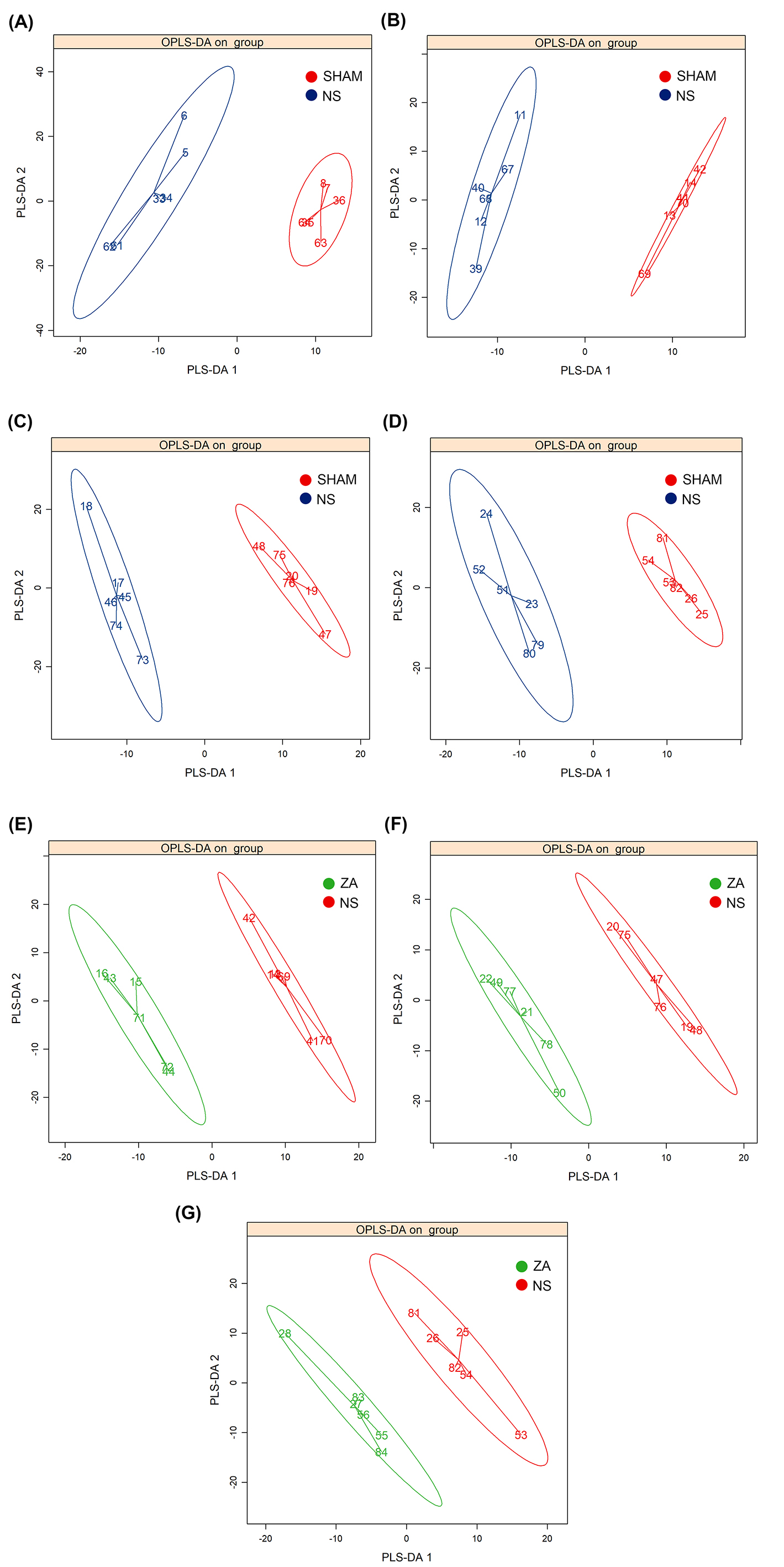


**Supplementary Figure S4.** PLS-DA score plots. NS versus SHAM at week 1 (A), week 6 (B), week 9 (C), and week 12 (D); ZA versus NS at week 6 (E), week 9 (F), and week 12 (G).


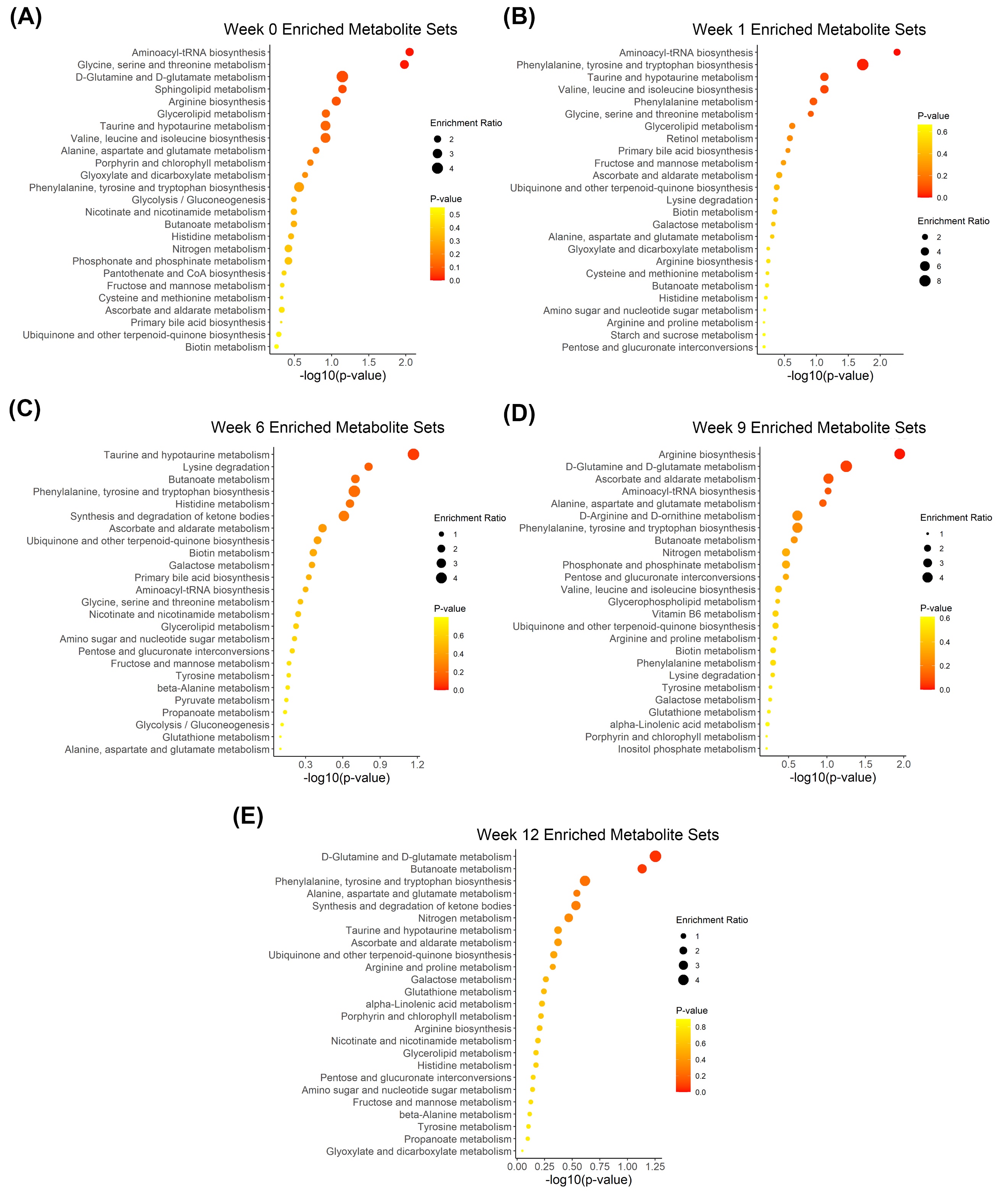


**Supplementary Figure S5.** Metabolic pathway enrichment analysis regarding OVX versus SHAM at week 0 (A); NS versus SHAM at week 6 (B), week 9 (C) and week 12 (D).

## Supplementary Tables

**Supplementary table S1.** The OPLS-DA model parameters of different groups.

| Group | Time | R^2^X | R^2^Y | Q2 |
| --- | --- | --- | --- | --- |
| OVX/NS  vs  SHAM | Week 0 | 0.369 | 0.990 | 0.768 |
|  | Week 1 | 0.386 | 0.983 | 0.739 |
|  | Week 6 | 0.282 | 0.994 | 0.786 |
|  | Week 9 | 0.357 | 0.989 | 0.720 |
|  | Week 12 | 0.357 | 0.982 | 0.745 |
| ZA vs NS | Week 1 | 0.186 | 0.992 | -0.068 |
|  | Week 6 | 0.296 | 0.992 | 0.569 |
|  | Week 9 | 0.252 | 0.991 | 0.293 |
|  | week1 2 | 0.270 | 0.979 | -0.449 |

**Supplementary table S2.** Intersections of differential metabolites (NS versus SHAM group)

| Compound Name | Week 0 | | | | Week 1 | | | | Week 6 | | | | Week 9 | | | | Week 12 | | | |
| --- | --- | --- | --- | --- | --- | --- | --- | --- | --- | --- | --- | --- | --- | --- | --- | --- | --- | --- | --- | --- |
|  | mean ratio | p | VIP | AUC | mean ratio | p | VIP | AUC | mean ratio | p | VIP | AUC | mean ratio | p | VIP | AUC | mean ratio | p | VIP | AUC |
| gamma-aminobutyrate (GABA) | 0.193 | 0.00507 | 2.02948 | 1 | 0.21 | 0.00824 | 2.12965 | 0.97222 | 0.124 | 0.00507 | 2.28798 | 1 | 0.31 | 0.02024 | 1.55284 | 0.91667 | 0.173 | 0.00824 | 1.99994 | 0.97222 |
| 2-aminobutyrate | 0.197 | 0.01307 | 1.96995 | 0.94444 | 0.193 | 0.00824 | 2.14664 | 0.97222 | 0.159 | 0.00507 | 2.25856 | 1 | 0.357 | 0.04533 | 1.43987 | 0.86111 | 0.182 | 0.00507 | 1.94628 | 1 |
| 2-hydroxybutyrate/2-hydroxyisobutyrate | 0.366 | 0.00824 | 1.86922 | 0.97222 | 0.302 | 0.00507 | 2.08536 | 1 | 0.32 | 0.00507 | 2.00485 | 1 | 0.379 | 0.02024 | 1.75981 | 0.91667 | 0.311 | 0.00507 | 1.9597 | 1 |
| taurocyamine | 0.492 | 0.00824 | 1.87519 | 0.97222 | 0.475 | 0.03064 | 1.68099 | 0.88889 | 0.438 | 0.00507 | 1.85488 | 1 | 0.462 | 0.03064 | 1.64156 | 0.88889 | 0.39 | 0.00824 | 1.86376 | 0.97222 |
| indolepropionate | 0.502 | 0.02024 | 1.71824 | 0.91667 | 0.507 | 0.00507 | 2.2345 | 1 | 0.443 | 0.00507 | 2.25104 | 1 | 0.347 | 0.00507 | 2.05067 | 1 | 0.42 | 0.00507 | 1.90605 | 1 |
| kynurenate | 0.438 | 0.00507 | 2.00771 | 1 | 0.541 | 0.00507 | 2.13341 | 1 | 0.371 | 0.00507 | 2.21871 | 1 | 0.471 | 0.00507 | 1.97256 | 1 | 0.45 | 0.01307 | 1.80033 | 0.94444 |
| indoleacrylate | 0.386 | 0.00507 | 1.88393 | 1 | 0.347 | 0.00507 | 2.23284 | 1 | 0.36 | 0.00507 | 2.32257 | 1 | 0.24 | 0.00507 | 2.18774 | 1 | 0.309 | 0.00507 | 2.06818 | 1 |
| thyroxine | 1.604 | 0.01307 | 1.73617 | 0.94444 | 1.584 | 0.00507 | 1.99451 | 1 | 1.39 | 0.03064 | 1.71818 | 0.88889 | 1.638 | 0.00507 | 2.11848 | 1 | 1.514 | 0.00824 | 1.8874 | 0.97222 |
| homoarginine | 0.354 | 0.00507 | 2.13524 | 1 | 0.614 | 0.03064 | 1.80033 | 0.88889 | 0.373 | 0.00507 | 2.31807 | 1 | 0.432 | 0.00507 | 2.04532 | 1 | 0.404 | 0.00507 | 2.00467 | 1 |
| N-monomethylarginine | 0.334 | 0.00824 | 1.86617 | 0.97222 | 0.503 | 0.00507 | 1.86281 | 1 | 0.581 | 0.00507 | 1.9919 | 1 | 0.589 | 0.00507 | 1.99952 | 1 | 0.577 | 0.00824 | 1.76878 | 0.97222 |
| N-acetylneuraminate | 0.679 | 0.02024 | 1.76938 | 0.91667 | 0.707 | 0.03064 | 1.6599 | 0.88889 | 0.545 | 0.01307 | 1.6433 | 0.94444 | 0.589 | 0.00507 | 2.24542 | 1 | 0.64 | 0.02024 | 1.8411 | 0.91667 |
| gulonate* | 0.526 | 0.03064 | 1.67136 | 0.88889 | 0.566 | 0.00824 | 1.94302 | 0.97222 | 0.496 | 0.00824 | 2.03982 | 0.97222 | 0.322 | 0.00507 | 2.1247 | 1 | 0.397 | 0.00507 | 2.04717 | 1 |
| linoleoyl-linolenoyl-glycerol (18:2/18:3) [1]* | 3.401 | 0.03035 | 1.50612 | 0.88889 | 3.568 | 0.00824 | 2.00536 | 0.97222 | 2.563 | 0.04533 | 1.67075 | 0.86111 | 5.163 | 0.00477 | 2.19359 | 1 | 2.076 | 0.0078 | 1.80751 | 0.97222 |
| linoleoyl-linolenoyl-glycerol (18:2/18:3) [2]* | 4.049 | 0.03064 | 1.65474 | 0.88889 | 3.007 | 0.00824 | 1.86267 | 0.97222 | 2.334 | 0.01307 | 1.75211 | 0.94444 | 4.534 | 0.00507 | 2.12487 | 1 | 3.284 | 0.01307 | 2.03343 | 0.94444 |
| linoleoyl-linoleoyl-glycerol (18:2/18:2) [1]* | 2.912 | 0.03064 | 1.4725 | 0.88889 | 2.442 | 0.01307 | 1.95749 | 0.94444 | 1.931 | 0.03064 | 1.67728 | 0.88889 | 2.843 | 0.00507 | 2.10146 | 1 | 2.417 | 0.01307 | 1.8214 | 0.94444 |
| oleoyl-linoleoyl-glycerol (18:1/18:2) [1] | 3.617 | 0.03064 | 1.56319 | 0.88889 | 2.696 | 0.02024 | 1.91994 | 0.91667 | 2.88 | 0.00824 | 2.08175 | 0.97222 | 4.575 | 0.00507 | 2.02782 | 1 | 3.692 | 0.00824 | 1.99029 | 0.97222 |
| oleoyl-linoleoyl-glycerol (18:1/18:2) [2] | 3.981 | 0.03064 | 1.58405 | 0.88889 | 3.005 | 0.02024 | 1.95487 | 0.91667 | 2.494 | 0.04533 | 1.75086 | 0.86111 | 5.057 | 0.00507 | 2.02131 | 1 | 3.538 | 0.00824 | 2.04221 | 0.97222 |
| palmitoleoyl-arachidonoyl-glycerol (16:1/20:4) [2]* | 5.839 | 0.01307 | 1.72398 | 0.94444 | 3.756 | 0.00824 | 1.89788 | 0.97222 | 3.001 | 0.00824 | 1.91776 | 0.97222 | 11.115 | 0.00507 | 2.15291 | 1 | 4.514 | 0.00507 | 2.0378 | 1 |
| 2S,3R-dihydroxybutyrate | 0.239 | 0.00824 | 1.96721 | 0.97222 | 0.202 | 0.00507 | 2.2195 | 1 | 0.182 | 0.00507 | 2.17128 | 1 | 0.329 | 0.04533 | 1.56404 | 0.86111 | 0.174 | 0.00507 | 2.01198 | 1 |
| 1-oleoylglycerol (18:1) | 3.545 | 0.02024 | 1.65338 | 0.91667 | 2.482 | 0.01307 | 1.94416 | 0.94444 | 3.582 | 0.01307 | 1.98727 | 0.94444 | 2.258 | 0.01291 | 1.99576 | 0.94444 | 3.2 | 0.00824 | 1.93287 | 0.97222 |
| 1-stearoyl-2-linoleoyl-GPE (18:0/18:2)* | 2.528 | 0.00824 | 1.77088 | 0.97222 | 1.721 | 0.02024 | 1.80359 | 0.91667 | 2.262 | 0.02024 | 1.75401 | 0.91667 | 2.026 | 0.02024 | 1.85578 | 0.91667 | 2.693 | 0.00507 | 2.01572 | 1 |
| 5,6-dihydrouridine | 0.666 | 0.01307 | 1.92418 | 0.94444 | 0.709 | 0.00507 | 2.34292 | 1 | 0.745 | 0.00507 | 2.02829 | 1 | 0.681 | 0.00507 | 2.17783 | 1 | 0.7 | 0.00507 | 1.98349 | 1 |
| pseudouridine | 0.771 | 0.01307 | 1.77941 | 0.94444 | 0.832 | 0.00824 | 2.07318 | 0.97222 | 0.863 | 0.00507 | 1.97993 | 1 | 0.82 | 0.00507 | 2.15308 | 1 | 0.784 | 0.00507 | 2.10395 | 1 |
| gamma-glutamyl-2-aminobutyrate | 0.288 | 0.03064 | 1.75923 | 0.88889 | 0.186 | 0.00507 | 2.30788 | 1 | 0.193 | 0.00507 | 2.26156 | 1 | 0.316 | 0.03064 | 1.45907 | 0.88889 | 0.141 | 0.00824 | 1.96982 | 0.97222 |
| 4-vinylphenol sulfate | 0.163 | 0.00507 | 2.09934 | 1 | 0.349 | 0.00507 | 2.06556 | 1 | 0.1 | 0.00507 | 2.09577 | 1 | 0.095 | 0.00507 | 2.04614 | 1 | 0.104 | 0.00824 | 2.00196 | 0.97222 |
| salicylate | 0.364 | 0.02024 | 1.78773 | 0.91667 | 0.546 | 0.01307 | 1.74722 | 0.94444 | 0.358 | 0.00824 | 1.97882 | 0.97222 | 0.225 | 0.00507 | 2.06265 | 1 | 0.413 | 0.00824 | 1.8164 | 0.97222 |

AUC, area under curve; * the metabolite was identified by searching online databases.
